# Symptom severity of bipolar disorder during the menopausal transition

**DOI:** 10.1186/s40345-015-0035-z

**Published:** 2015-08-22

**Authors:** Wendy K. Marsh, Bernice Gershenson, Anthony J. Rothschild

**Affiliations:** Department of Psychiatry, School of Medicine, University of Massachusetts, 55 Lake Ave North, S3-314, Worcester, MA 01655 USA; Department of Orthopedics and Physical Rehabilitation, University of Massachusetts, 55 Lave Ave N - AC7 069, Worcester, MA USA; Department of Psychiatry, University of Massachusetts, 55 Lave Ave North, Worcester, MA USA

**Keywords:** Bipolar disorder, Menopause, Neuroendocrinology, Estrogen

## Abstract

**Background:**

Little is known about the mood symptom experience of women with bipolar disorder during the menopausal transition (MT). Yet times of rapid hormonal decline, such as the postpartum, are associated with increased risk of severe mood episodes in bipolar disorder, and the MT is a time of increased risk for unipolar depression in women with or without a history of depression.

**Methods:**

Enrollment included 56 women 40–60 years old diagnosed in the bipolar spectrum who were experiencing menopausal symptoms or were up to 5 years since their final menstrual period. Menopausal stages included early menopause, late menopause, or early postmenopause based on standardized criteria. Observational, prospective standardized mood symptom and reproductive hormone assessments were completed periodically. Concurrent menopausal symptoms as well as history of mood exacerbation during past reproductive events were assessed.

**Results:**

Forty-four women were included in the main analysis. The average Montgomery-Asberg Depression Rating Scale (MADRS) score was 4.43 points higher in the late transition/early postmenopausal stage women (*n* = 29) compared to the early menopausal stage women (*n* = 15) (±SE 2.14; *p* = 0.039), corresponding to a roughly 10 % higher score (range 0–40) in the late/post stage across all study visits. Results were similar for the Young Mania Rating Scale (YMRS), where the average score was 2.54 points higher in the late/early postmenopausal stage women compared to the early menopausal stage women (±SE 1.15; *p* = 0.027), also roughly 10 % higher (range 0–26). Estradiol and follicle-stimulating hormone (FSH) absolute levels as well as between-visit change in levels were not notably associated with YMRS or MADRS during study observation. Total Greene Climacteric Symptom (menopausal symptom) score was significantly associated with MADRS but not YMRS. History of mood exacerbation premenstrually and/or postpartum was not significantly associated with YMRS or MADRS severity during the MT.

**Conclusions:**

These results support the theory that times of increased reproductive hormonal changes, such as the late MT and early postmenopause, here compared to early MT, are associated with greater mood symptom severity in bipolar spectrum women. Nonetheless, absolute or change in FSH and estradiol levels were not significantly associated with depression or mood elevation severity.

## Background

Due to the scarcity of studies, little is known about the course of bipolar disorder through the perimenopause, or menopausal transition (MT), and yet every woman with bipolar disorder expects to or has experienced menopause. Compelling evidence on the increased risk of mood episodes in women with bipolar disorder (BD) during other times of reproductive hormonal transition, like postpartum (Kendell et al. 1987; Sit et al. 2006), indicates the importance of examining mood during the menopausal transition. Given the substantial societal burden of bipolar disorder (Kleine-Budde et al. 2013), detecting times of risk is critical.

Psychological symptoms in women without a mood disorder are frequent during the MT and most often include irritability, tearfulness, anxiety, depression, emotional lability, low energy and motivation, poor concentration, and interrupted sleep (Avis et al. 1994). While the role of reproductive hormones in the risk of mood symptoms is unclear, psychological symptoms are postulated to be linked to the fluctuation, not absolute levels, of estradiol (Rubinow et al. 1998). These psychological symptoms overlap with those of functionally impairing mood disorders leading one to speculate that the perimenopause may contribute to depression or mood elevation in vulnerable women.

Unipolar depression risk in the MT has an expanding literature reporting an increase of both major depression and depressive symptoms during the perimenopause (Maartens et al. 2002; Freeman et al. 2004a; Schmidt et al. 2004; Bromberger 2006; Cohen et al. 2006; Steinberg et al. 2008). Compared with premenopausal women, women with a history of depression are nearly five times more likely to have a diagnosis of major depression in the menopausal transition, whereas women with no history of depression are two to four times more likely to report depressed mood (Freeman 2010). Within the Stages of Reproductive Aging Workshop + 10 (Harlow et al. 2012) defined reproductive stages, the late perimenopause (interval of amenorrhea of 60 to 364 days) (Dennerstein et al. 1993; Schmidt et al. 2004; Freeman et al. 2006; Bromberger et al. 2007; Woods et al. 2008; Freeman 2010; Bromberger et al. 2011) and early postmenopause (amenorrhea 1–6 years) (Dennerstein et al. 2004; Bromberger et al. 2007; Bromberger et al. 2011) are times of heightened risk of unipolar depression. Women reporting a history of premenstrual or postpartum mood disturbance are at greater risk of depression during the MT than those who do not (Freeman et al. 2004b; Freeman 2010).

Less is known in bipolar disorder. Postmenopausal women recalling the MT report severe mood disturbances or worsening depression during the transition (Blehar et al. 1998; Freeman et al. 2002). Mood elevation is reported less frequently than depression in the MT (Blehar et al. 1998; Kennedy et al. 2005). Our work reviewing longitudinal standardized clinical assessments in menopausal age women with bipolar disorder found a high degree of depression, 68 % over a 17-month average duration, that was significantly greater than what subjects reported experiencing during their reproductive years (Marsh et al. 2008). Compared to a concurrent pooled comparison group of like-aged men and reproductive age women with bipolar disorder, women of menopausal age with bipolar disorder had a significantly greater proportion of clinic visits in the depressed state and significantly lower proportion in the euthymic state, with no difference in proportion of visit in the elevated/mixed state (Marsh et al. 2009). When examining mood by menopausal stage in a large multisite database of patients treated for bipolar disorder, the small number of women transitioning from perimenopause to postmenopause had significantly greater depression than other female reproductive groups while overall euthymia and mood elevation decreased with progressing female reproductive stage (Marsh et al. 2012). Nonetheless, there is a lack of prospective data on mood experience during this critical time in bipolar disorder.

This study prospectively examines mood across the MT stages and in association with reproductive hormones in women with bipolar disorder. We hypothesized that depression scores would be higher during the late menopausal transition and at times of greater hormonal changes.

## Methods

### Study participants

Study enrollment occurred from January 2010 to July 2012, and subjects were recruited from the community as well as from an academic psychiatry clinic. Eligibility included (a) a diagnosis of bipolar disorder (I, II, NOS) based on DSM IV criteria, (b) age 40–60 years with intact uterus and ovaries, (c) a menstrual period within the last 5 years, if menstruating regularly (every 21–35 days) subject must be experiencing menopausal symptoms, and (d) having a current psychiatric provider. Menopausal symptoms were defined narrowly as vasomotor symptoms (hot flashes and night sweats) so as not to overlap with mood symptoms (for example, sleep disturbance and irritability). As study personnel did not assume care of subjects, for subject safety, a psychiatric provider was required. Women using vaginal hormone therapy (*n* = 1) or intrauterine device with hormonal progesterone (*n* = 1) were accepted; those on oral contraceptives were not. Participants provided signed, written informed consent prior to study entry.

### Procedures

This study conducted an observational longitudinal evaluation of mood for 4 months during each of the following menopausal stages: late reproductive, early menopausal transition, late menopausal transition, and early postmenopause (defined in the “Menopausal status” section below) in women with bipolar disorder. The initial interview was scheduled in the early follicular phase on days 2–6 of the menstrual cycle (day 1 being the first day of menstruation) in women who were menstruating more frequently than every 60 days for standardized hormonal assessments. Initial visit included a standardized interview with the Affective Disorder Evaluation (ADE), a modified version of the mood and psychosis modules from the Structured Clinical Interview for DSM Disorders (SCID) intended for routine use by practicing clinicians, which was performed by an ADE trained psychiatrist. The ADE provided diagnoses of DSM IV bipolar I disorder, bipolar II disorder, bipolar disorder not otherwise specified, psychiatric and medical comorbidities, as well as mood disorder history (Sachs et al. 2003). The ADE includes standardized questions for subject report of the percent of time in the prior 12 months spent experiencing mood elevation, depression, or anhedonia symptoms. It also asks for patient self-reported endorsement of a history of premenstrual or postpartum mood exacerbation. Assessments of current mood state severity with Young Mania Rating Scale (YMRS) (Young et al. 1978) and Montgomery-Asberg Depression Rating Scale (MADRS) (Montgomery and Asberg 1979) were performed by two trained raters who periodically compared score results for inter-rater reliability. Subjects completed self-administered questionnaires detailing menstrual and medical history and the Greene Climacteric Scale (Greene 1976) to assess menopausal symptoms. A blood sample was obtained in the early follicular phase of the menstrual cycle to assess follicle-stimulating hormone (FSH) and estradiol levels. In women who had not menstruated for 60 days or longer, a random hormonal sample was assessed.

Follow-up visits included standardized YMRS and MADRS mood assessments. If a woman was menstruating with predictability, visits would be scheduled up to 6 weeks apart in the early follicular phase (days 2–6) of her menstrual cycle and hormone blood draws performed. If a woman had gone 60 days without menstruation at intake visit, then mood assessments were scheduled once a month and hormone blood draws were scheduled every other month regardless of where a woman was in her menstrual cycle. The study was approved by the University of Massachusetts Medical School Institutional Review Board.

### Serum hormone measures

Estradiol and follicle-stimulating hormone were analyzed by institutional standard laboratory procedures. Estradiol levels were assessed by Access Estradiol, a competitive binding immune-enzymatic assay. FSH was assayed by Access hFSH which is a sequential two-step immune-enzymatic assay.

### Menopausal status

Menopausal staging was determined using the executive summary of Staging of Reproductive Aging in Women (STRAW) designed as a comprehensive basis for assessing reproductive aging in research settings (Harlow et al. 2012). Subjects were categorized based on STRAW + 10 stages: (1) second half of the late reproductive (subtle changes in menstrual cycle characteristics and vasomotor symptoms), (2) early perimenopasue (increased variability in menstrual cycle length defined as a persistent difference of 7 or more days in the length of consecutive cycles), (3) late perimenopause (no menstrual cycle in 60 days but menstrual bleeding in the last 12 months), and (4) early postmenopause (no menstrual cycle in the last 1–5 years). Given the small sample size, the reproductive groups were collapsed.

As hormonal levels and menstrual cyclicity remain relatively predictable in the earlier stages of the MT (Harlow et al. 2012), the late reproductive and early MT women were collapsed into one group, called early transition. Hormonal levels show greater variability and unpredictability during the later menopausal transition stages, including the early postmenopause (Burger et al. 1999; Burger et al. 2007; Sowers et al. 2008a; Sowers et al. 2008b; Harlow et al. 2012). In accordance, the late perimenopausal and early postmenopausal women were collapsed into one group, called the late and early-post transition group.

### Statistical analysis

The two groups analyzed included (a) the early transition group, consisting of women in late reproductive (*n* = 4) or early perimenopause (*n* = 17) phase, and (b) the late and early-post transition, women in their late perimenopause (*n* = 20) or early postmenopause (*n* = 15) phase.

Chi-square (*χ*^2^) tests were used to compare categorical variables, and two sample *t* tests with equal variance were utilized to test differences in continuous variables by the two groups; Pearson correlation coefficients measured the linear association between two variables (Sedgwick 2012). For the main outcome measure, we analyzed longitudinal changes across visits for MADRS and YMRS sums using generalized estimating equation (GEE) methods to control for the correlation within the subjects at the five study time points. Models were run examining potential associations between MADRS and YMRS and menopausal stage categories (early vs. late/post), adjusted for study visit (where visit 1 was used as the referent category). We used an autoregressive covariance structure in all of our GEE models. *p* values are reported from a *z* test that a single regression coefficient was equal to 0. We also tested the significance of difference among the time effects as a multiple degrees of freedom chi-square test. SAS software version 9.3 (SAS Institute, Cary, NC) was used for the GEE modeling while SAS version 9.2 was used for the remaining analyses.

## Results

Of 255 women screened, 99 were eligible and 57 enrolled. One woman was dropped due to active illicit substance abuse influencing mood at the time of enrollment. Of the 56 women who entered the study, 21 % (*n* = 12) completed only the first visit, 32 % (*n* = 18) completed two visits, and 66 % (*n* = 37) completed all third, fourth, and final visits. The dropout rate in the early transition group was 43 % and the late and early-post transition group 31 %. There were no significant differences in demographic characteristics, bipolar disorder characteristics, or treatment approach between the early transition and the late and early-post transition groups with the exception of age-dependent variables (age and duration of illness) being greater in the older aged late and early-post transition group. Current alcohol use not meeting the criteria for dependence or abuse was more common in the early transition group. There was no significant difference between reproductive groups in other common comorbidities that have been shown to affect mood course in BD including rapid cycling status, age at onset (Schurhoff et al. 2000), current comorbid anxiety (Simon et al. 2004), and substance use (Goldstein et al. 2006) disorders. The sample characteristics are presented in Table 1.

**Table 1:** Subject characteristics

	Early MT (*n* = 21)	Late MT/early postmenopause (*n* = 35)	*p* value	Total (*n* = 56)
Age, mean ± SD	45.0 ± 3.4	50.6 ± 4.7	<0.001	48.5 ± 5.0
Age at onset of bipolar disorder, mean ± SD	14.9 ± 7.1	15.5 ± 8.6	0.82	15.3 ± 8.0
Bipolar diagnosis, *N* (%)			0.45	
I	11 (53)	17 (48)		28 (50)
II	7 (33)	16 (45)		23 (41)
NOS	2 (14)	2 (6)		5 (9)
Duration of illness, mean ± SD	30.0 ± 6.7	35.1 ± 8.6	0.02	33.2 ± 8.3
Rapid cycling, *N* (%)	13 (61.9)	24 (75)	0.31	37 (69)
Ethnicity, Caucasian, non-Hispanic, *N* (%)	17 (80)	33 (97)	0.18	50 (91)
Marital status, married/partnered, *N* (%)	10 (48)	19 (56)	0.55	29 (53)
Employment status, full or part time, *N* (%)	8 (38)	14 (41)	0.82	22 (40)
BMI, mean ± SD	28.4 ± 5.7	31.3 ± 7.3	0.14	30.1 ± 6.8
Comorbid anxiety, *N* (%)	19 (90)	33 (94)	0.60	52 (93)
Comorbid alcohol use, *N* (%)	8 (38)	2 (6)	<0.01	10 (18)
Comorbid substance use, *N* (%)	3 (14)	8 (23)	0.43	11 (20)
Number of mood stabilizers (Li, AED, and antipsychotics), mean ± SD	1.28 ± 0.8	1.25 ± 0.7	0.88	1.27 ± 0.7

### Menopausal stage and mood

One woman who entered the study in late menopause transitioned to early postmenopause during the study observation. One woman who entered the study in early menopause proceeded to late menopausal transition during the study observation; she was analyzed in the early transition group as the diagnosis of change of reproductive stage is made after the occurrence, as it would in clinical practice.

A total of 44 women were included in this analysis, as they had information from at least two visits in order to adjust for the autocorrelation within women. Of these women, 15 were in the early transition group and 29 were in the late and early-post transition (5 of which were over the age of 55).

Results from the MADRS model showed that average MADRS score was 4.43 points higher in the late and early-post transition stage women compared to the early transition women (±SE 2.14; *p* = 0.039), corresponding to a roughly 10 % increase in score (score range 0–40) in the late/post stage across all study visits. The effect of time on the MADRS score was not significant (*χ*^2^ = 9.41; *df* = 4; *p* = 0.052). The mean MADRS score for the early transition women was 12.10 (±SE 1.63), 95 % CI 8.89, 15.30, and for the late and early-post transition women, the mean was 16.52 (±SE 1.67) and 95 % CI 13.25, 19.80.

Results were similar for the YMRS model, where average score was 2.54 points higher in the late and early-post transition women compared to the early transition women (±SE 1.15; *p* = 0.027), also a roughly 10 % increase (score range 0–26). The effect of time on the YMRS score was not significant (*χ*^2^ = 8.68; *df* = 4; *p* = 0.07). The mean YMRS score for early transition women was 6.06 (±SE 1.02), 95 % CI 4.07, 8.06, and for late and early-post transition women, the mean was 8.60 (±SE 1.02), 95 % CI 6.60, 10.60.

In contrast, retrospective report of percent of past year in depressed/anhedonic or elevated mood state was not associated with menopausal stage (Table 2).

**Table 2:** Bipolar depression and mood elevation reported in the previous year by menopausal stage

	Menopausal stage		
	Early MT (*n* = 21)	Late MT/early postmenopause (*n* = 35)	*p* value
% of previous year reported in a depressed mood	36.3 ± 32.1	39.5 ± 26.2	0.69
% of previous year reported in an elevated mood	10.3 ± 11.6	16.5 ± 17.5	0.16

### Neuroendocrine results

Mean FSH increased and mean estradiol decreased as would be expected across the progression from early to later menopausal transition to postmenopause (Table 3). Estradiol levels varied by 264 pg/ml (19–283 pg/ml) for FSH levels less than 40 IU/L; for FSH levels greater than or equal to 40 IU/L, estradiol levels remained low <44 IU/L, in accordance with the clinically accepted FSH cutoff of 40 IU/L.

**Table 3:** Reproductive hormone levels by menopausal stage

		FSH (IU/L)		Estradiol (pg/ml)	
	*N*	Mean ± SD	*p* value	Mean ± SD	*p* value
Early MT	20	11.6 ± 11.4	<0.0001	74.4 ± 63.8	=0.09
Late MT	19	53.1 ± 47.6		53.8 ± 69.3	
Early postmenopause	14	79.9 ± 33.2		27.9 ± 26.3	

At initial visit, there was a positive correlation between absolute estradiol level and YMRS (*r*(51) = 0.31, *p* = 0.02) across reproductive groups. This significant association was not born out when estradiol levels and YMRS scores were assessed at follow-up visits. FSH levels were not associated with YMRS score at intake or follow-up visits. Neither FSH nor estradiol absolute levels were associated with MADRS scores at any visit. Likewise, the amount of change in FSH or estradiol levels from the previous visit to the assessed visit was not associated with MADRS of YMRS score at any follow-up visit (Table 4).

**Table 4:** Bipolar depression and mood elevation score correlation with between-visit change in hormone levels

Visit	*N*	MADRS and change in FSH	MADRS and change in estradiol	YMRS and change in FSH	YMRS and change in estradiol
1–2	*24*	*r*(22) = 0.22 *p* = 0.29	*r*(22) = 0.22 *p* = 0.30	*r*(22) = −0.01 *p* = 0.97	*r*(22) = 0.03 *p* = 0.90
2–3	16	*r*(14) = −0.09 *p* = 0.72	*r*(14) = 0.16 *p* = 0.56	*r*(14) = −0.14 *p* = 0.6	*r*(14) = 0.29 *p* = 0.28
3–4	*19*	*r*(17) = −0.20 *p* = 0.41	*r*(17) = 0.02 *p* = 0.92	*r*(17) = −0.29 *p* = 0.22	*r*(17) = 0.34 *p* = 0.16
4–5	*18*	*r*(16) = 0.15 *p* = 0.54	*r*(16) = 0.01 *p* = 0.97	*r*(16) = 0.20 *p* = 0.43	*r*(16) = −0.40 *p* = 0.10

### Menopausal symptoms and mood

Mean total Greene Climacteric Symptom rating was 23.4 (SD ± 10.7) in line with mean of 22.9 of normative results of 40–55-year-old women visiting a menopausal clinic. Greene score was significantly associated with concurrent MADRS score, *r*(52) = 0.52, *p* < 0.001, but not YMRS score at baseline visit. On sub-scale analysis, MADRS score was associated with Greene Psychological sub-scale, *r*(52) = 0.55, *p* < 0.001, but not with Greene Vasomotor subscale (Table 5).

**Table 5:** Greene Climacteric Symptom scale overall and subsection correlation with bipolar depression and mood elevation scores

Greene	MADRS *N* = 54	YMRS *N* = 54
Overall	*r*(52) = 0.52	*r*(52) = 0.06
	*p* < 0.0001	*p* = 0.66
Subsection		
Somatic	*r*(52) = 0.24	*r*(52) = 0.19
	*p =* 0.09	*p* = 0.21
Vasomotor	*r*(52) = 0.19	*r*(52) = −0.22
	*p* = 0.16	*p* = 0.23
Psychological	*r*(52) = 0.55	*r*(52) = 0.08
	*p* < 0.001	*p* = 0.59

### History of reproductive events and mood disruption

Women who had given birth (*n* = 36) and reported no postpartum mood exacerbation tended to have lower, although not statistically significant (*t* = −1.91, *p* = 0.07), mean YMRS across visits (7.2 ± 7.0) than those who reported postpartum mood exacerbations (YMRS 11.2 ± 5.5). Otherwise, reported history of a postpartum and or perimenstrual mood exacerbation was not associated with mean severity of MADRS or YMRS in the MT and early postmenopausal years (Table 6).

**Table 6:** Difference in bipolar depression and mood elevation scores during the menopausal transition based on history of reproductive phase mood exacerbation

Reproductive group	Reproductive phase mood exacerbation (by woman), *N* (%)	Mean MADRS ± SD	*t* test	*p* value	Mean YMRS ± SD	*t* test	*p* value
Perimenstrual							
*N*_women_ = 55							
History of perimenstrual mood exacerbation	47 (85)	15.4 ± 8.5	*t* = 0.20	*p* = 0.85	9.0 ± 6.1	*t* = −0.59	*p* = 0.56
No history of perimenstrual mood exacerbation	8 (15)	16.1 ± 14.8			7.6 ± 7.2		
Postpartum							
*N*_women_ = 46							
History of postpartum mood exacerbation	23 (64)	14.4 ± 7.9	*t* = 0.33	*p* = 0.74	11.2 ± 5.5	*t* = −1.91	*p* = 0.07
No history of postpartum mood exacerbation	13 (36)	15.5 ± 10.8			7.2 ± 7.0		
Perimenstrual and postpartum							
*(N*_women_ = 46)							
Both: history of postpartum and perimenstrual mood exacerbation	20 (91)	14.3 ± 8.0	*t* = 0.97	*p* = 0.34	10.9 ± 5.8	*t* = −0.75	*p* = 0.46
Neither: no history of postpartum or perimenstrual mood exacerbation	2 (9)	21.0 ± 22.6			7.5 ± 10.6		

## Discussion

The primary finding of this study is significantly higher depression and mood elevation symptom scores in women with bipolar disorder during the late menopausal transition and early postmenopause than those of women in the late reproductive years and early menopausal transition. A difference of greater than two points on the MADRS (4.4 points in this report) has been called clinically meaningful (Kennedy et al. 2006). A YMRS difference of 2.5 points reported in this study, even in an outpatient population, while statistically significant, is less likely to be clinically significant (Lukasiewicz et al. 2013).

The increased level of depressive symptoms in the late and early-post transition group is consistent with previous chart reviews (Marsh et al. 2012) and retrospective recall (Blehar et al. 1998; Freeman et al. 2002) of the MT in women with bipolar disorder. It is also in agreement with the greater rates, compared to premenopause, of depression during the late MT (Schmidt et al. 2004; Freeman 2010; Freeman et al. 2014) and often early postmenopause (Bromberger et al. 2010) found in the unipolar depression literature. The statistically significant higher YMRS mood elevation rating in the late and early-post transition group than early transition group is in contrast to the decrease in DSM IV mood elevation symptoms reported previously (Marsh et al. 2012) but does not contradict the retrospective report of worsening mood elevation or irritability retrospectively reported by many postmenopausal women with bipolar disorder (Blehar et al. 1998; Freeman et al. 2002). The concomitant worsening of both depression and mood elevation symptoms in the later stages of the menopausal transition may be indicative of worsening of mixed features, now a DSM V specifier for bipolar disorder (APA 2013).

Given these early results, future studies are needed, particularly with a larger sample size to confirm, or refute, the menopausal transition as a time of risk of depression, mood elevation or mixed symptoms in the course of bipolar disorder. Larger studies would further elucidate the role of clinically defined menopausal stage by evaluating late reproductive, early transition, late transition, early postmenopause, and late postmenopause. A longer duration of observation would ideally include observing mood across menopausal stages within individual subjects. Of particular interest would be the role of the last menstrual period as a marker in the late transition and early postmenopause (Freeman et al. 2014) and as an easy reference point in the clinical discussion with a patient.

### Neuroendocrine

This is the first study to examine reproductive hormones in the MT in bipolar disorder. Despite a positive correlation between absolute estradiol level and YMRS at the initial visit, there were no further associations between absolute or change between visits in FSH or estradiol levels with either depression or mood elevation scores. While potentially a chance association of estradiol and mood elevation symptoms, this is the first report on estradiol levels and mood elevation. This result may indicate a subgroup of women who are vulnerable to mood elevation symptoms during times of high estradiol. No standard testing exists for assessing the degree of variability in hormonal levels after cessation of predictable menses, thus a potential future definition of variability, may yet reveal associations with mood severity.

Neurocognitive evidence suggests that estradiol may have antidepressant-like properties in the brain (Kendall et al. 1982; McEwen et al. 1997), and clinical trial results have reported an antidepressant effect of estradiol administration during the MT in unipolar depression (Soares et al. 2001; Rasgon et al. 2002). Nonetheless, results in unipolar depression during the MT overall do not relay an association of depressive symptoms with absolute FSH or estradiol levels (Schmidt et al. 2004; Bromberger et al. 2010) with the exception of increased FSH being positively associated with depressive symptoms in one study (Freeman et al. 2006). Reports on estradiol and FSH variability and risk of depression during the MT have been both positive (Freeman et al. 2004a; Freeman et al. 2006; Freeman et al. 2014) and negative (Schmidt et al. 2004; Bromberger et al. 2010; Freeman et al. 2014) and have employed differing ways of assessing variability.

More thorough hormonal assessments should include testosterone which has been found associated with unipolar depression during the MT (Bromberger et al. 2010), inhibin B, an early indicator of ovarian aging, and potentially sex hormone-binding globulin as well as dehydroepiandrosterone which have both been assessed in association with unipolar depression during the menopausal transition (Bromberger et al. 2010).

### Greene Climacteric Scale and vasomotor symptoms

This is the first evaluation of bipolar mood symptoms and menopausal symptoms. The Greene Climacteric Scale overall score was positively associated with concurrent depressive symptoms in women with bipolar disorder during the MT. This was driven by the Greene psychological subscale association with MADRS depression scores. MADRS depression scores in women with bipolar disorder were not associated with concurrent vasomotor symptoms reported on the Greene Scale. While the mechanism behind VMS is not well understood, an association between vasomotor symptoms and unipolar depressive symptoms has often (Cohen et al. 2006; Freeman et al. 2009) but not always been reported (Freeman 2010)*.*

The Greene Scale overall and subscale scores were not associated with mood elevation symptoms. Thus, despite the supposition that hormonal changes during the transition are behind VMS and the increased risk of unipolar depression as well as the overlap in menopause and mood symptoms (example sleep changes), the association of menopausal and mood symptoms did not bear out in bipolar disorder. As this is a cross-sectional report, it may be useful to further examine the duration of VMS or menopausal symptoms and risk of mood symptoms and assess for the potential of a longitudinal association.

### History of reproductive-related mood exacerbations

Women who reported a mood exacerbation during the postpartum period tended to experience more mood elevation symptoms during the MT than women who did not have a mood exacerbation in the postpartum. Depression scores in the MT did not significantly differ when a woman reported history of postpartum and or perimenstrual mood exacerbation. Likewise, mood elevation symptoms in the MT did not significantly differ if a woman reported a history of perimenstrual mood changes or perimenstrual and postpartum mood exacerbations.

A case series reports postpartum mood episodes associated with an increased risk of perimenopausal mood episodes in bipolar disorder (Robertson Blackmore et al. 2008). Ours and other previous work in bipolar disorder, in line with this study results, did not find reporting a menstrual cycle or postpartum mood exacerbation associated with perimenopausal depression (Payne et al. 2007; Marsh et al. 2012). In the unipolar depression literature, the results are mixed. Women, in one study reporting premenstrual symptoms, were at greater risk of menopausal depressed mood (Freeman et al. 2004b); however, this was not the case in a cross-sectional report in unipolar depression during the perimenopause (Steinberg et al. 2008).

The strength of this study is that it is the first to prospectively evaluate mood during the MT in women with bipolar disorder. The assessment of reproductive hormonal levels and mood in women with bipolar disorder has also not been reported prior. Likewise, VMS in women with bipolar disorder had not been previously reported in relation to mood. The limitations of this study include a small sample size due to higher than anticipated dropout and slow recruitment. The sample was too small to further evaluate early MT, late MT, and early postmenopause as separate groups. This study is also unable to differentiate what part, if any, the worse mood ratings in the older aged late and early post transition group may be due to worsening course of bipolar disorder with longer duration of the mental illness. Life stressors were not assessed.

## Conclusions

In summary, women with bipolar disorder were found to have significantly more depression and mood elevation symptoms during the late MT and early postmenopause compared to late reproductive age and early MT women. Overall, absolute levels and variability in FSH and estradiol were not associated with concurrent depression or mood elevation mood ratings despite an initial positive association of estradiol level and YMRS score at first visit. Menopausal symptoms, but not specifically VMS, were associated with concurrent depression but not mood elevation severity. A history of postpartum mood exacerbations may be associated with greater mood elevation symptoms during the MT. Further studies of greater duration are needed to discern whether the final menstrual period is pivotal in mood pattern in the late MT to early postmenopausal years. Knowing that, greater mood severity symptoms occurred during times of greater hormonal variability during the menopausal transition (the late MT and early postmenopause) may offer an opportunity to examine novel hormonal approaches to mood stability in women with bipolar disorder.
